# Neuroprotective Properties of Green Tea (*Camellia sinensis*) in Parkinson’s Disease: A Review

**DOI:** 10.3390/molecules25173926

**Published:** 2020-08-27

**Authors:** Dicson Sheeja Malar, Mani Iyer Prasanth, James Michael Brimson, Rajasekharan Sharika, Bhagavathi Sundaram Sivamaruthi, Chaiyavat Chaiyasut, Tewin Tencomnao

**Affiliations:** 1 Age-Related Inflammation and Degeneration Research Unit, Department of Clinical Chemistry, Faculty of Allied Health Sciences, Chulalongkorn University, Bangkok 10330, Thailand; sheeja.malar@gmail.com (D.S.M.); prasanth.m.iyer@gmail.com (M.I.P.); james.b@chula.ac.th (J.M.B.); 2 309, Vrinda, 10th Cross, Railway Layout, Vijayanagar 2nd Stage, Mysuru, Karnataka 570016, India; sharikarpillai@gmail.com; 3 Innovation Center for Holistic Health, Nutraceuticals and Cosmeceuticals, Faculty of Pharmacy, Chiang Mai University, Chiang Mai 50200, Thailand; sivasgene@gmail.com (B.S.S.); chaiyavat@gmail.com (C.C.)

**Keywords:** neurodegenerative diseases, Parkinson’s disease, green tea, polyphenols, neuroprotection

## Abstract

Neurodegenerative disease is a collective term given for the clinical condition, which results in progressive degeneration of neurons and the loss of functions associated with the affected brain region. Apart from the increase in age, neurodegenerative diseases are also partly affected by diet and lifestyle practices. Parkinson’s disease (PD) is a slow onset neurodegenerative disorder and the second most common neurodegenerative disease, which affects the motor system. Although there is no prescribed treatment method to prevent and cure PD, clinical procedures help manage the disease symptoms. Green tea polyphenols are known for several health benefits, including antioxidant, anti-inflammatory, and neuroprotective activity. The current manuscript summarizes the possible mechanisms of neuroprotective potential of green tea with a special focus on PD. Studies have suggested that the consumption of green tea protects against free-radicals, inflammation, and neuro-damages. Several in vivo studies aid in understanding the overall mechanism of green tea. However, the same dose may not be sufficient in humans to elicit similar effects due to complex physiological, social, and cultural development. Future research focused on more clinical trials could identify an optimum dose that could impart maximum health benefits to impart neuroprotection in PD.

## 1. Introduction

The tea plant *Camellia sinensis*, which grows in tropical and temperate regions of Asia, has been closely associated with humans. Initially, the leaves were chewed, pickled, or eaten as a vegetable. Historically an infusion from the plant was used in medicine as a relaxant, detoxifying agent, and for curing stomach problems, headache, and nervous tension [1,2].

**Figure 1:**
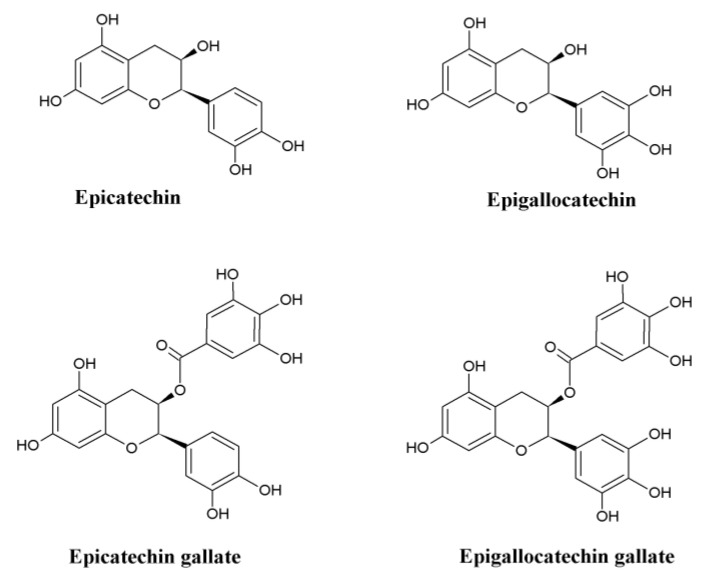
Chemical structures of major catechins in green tea.

Subsequently, rather than being used as a medicine, it became one of the most sought-after non-alcoholic beverages globally and is cultivated in more than 20 countries [3]. Though literature hints that green tea consumption has taken place for thousands of years, the search for phytochemicals responsible for the aroma and its medicinal properties started only in the early 1900s. Epicatechin, epicatechin gallate, epigallocatechin, and epigallocatechin gallate were identified as the major catechins in green tea (Figure 1). As the identification of phytochemicals emerged, the study on the role of green tea and its constituents in counteracting a diverse range of disease conditions also started to evolve along with the advancements in science. Consumption of green tea can induce many beneficial effects, including anti-aging, anti-photoaging, stress resistance, neuroprotection, and autophagy, to name a few [4]. This review mainly focusses on Parkinson’s disease (PD) and the molecular mechanism behind the neuroprotective action of green tea and its constituents against the disease. Consumption of green tea could have a possible role in modulating PD as it has been observed to mediate the known markers of PD in both in vitro and in vivo models, which is explained in the following sections.

## 2. Neurodegenerative Diseases

Neurodegenerative disease is a collective term given to the clinical conditions which result in progressive degeneration of neurons coupled with the loss of functions associated with the affected brain region. Some of the major neurodegenerative diseases other than PD include Alzheimer’s disease (AD), Huntington’s disease (HD), and prion diseases, which results in decreased cognitive abilities and interfering with the daily activities of the individuals living with neurodegenerative illness. The incidence of most of the neurodegenerative diseases increases with age and is considered multifactorial. Apart from the increase in age, the occurrence of neurodegenerative diseases is also partly affected by diet and lifestyle practices [5]. Although there have been several studies in identifying drugs for the treatment of neurodegenerative diseases, one of the significant challenges still faced by the researchers is the inability to regenerate neurons when they are lost [6].

## 3. Parkinson’s Disease

Parkinson’s disease is a slow onset neurodegenerative disorder that affects the motor system. PD usually affects people over the age of 50; when those under 50 are affected, it is usually referred to as early-onset PD. The disease gets its name from the English physician Dr. James Parkinson, who first described in detail the symptoms in the essay “An essay on the shaking palsy” [7] originally published in 1817, and reprinted in 2002 in the journal of neuropsychiatry and clinical neurosciences.

### 3.1. Symptoms

The initial signs of PD are often difficult to discern from healthy aging. Symptoms commonly associated with PD include tremors, stiffness, and difficulty in walking. The symptoms of PD can be divided into two groups; the motor symptoms and the neuropsychiatric symptoms. The most common symptom that first occurs in PD is a slow tremor in the hand while at rest, which disappears when the hand is voluntarily moved. As the disease progresses, the tremor often eventually affects both hands. The slowness of movement (bradykinesia) is found in every PD case and is one of the cardinal symptoms of PD [8]. Individuals with PD may suffer difficulty in movements such as those required to get up from a chair. In the early stages, the body stiffness is often asymmetrical, although as the disease progresses, both sides of the body are affected, which translates to difficulty in performing everyday functions. Bradykinesia may appear unpredictably, leading to frustration in the individuals as suddenly they are unable to perform a task that just minutes before was possible [9]. Stiffness and the loss of autonomic control result in a stopped posture and rigidity, causing a change in gait referred to as the parkinsonian gait, which is characterized by small shuffling steps with difficulty to start walking and stopping again. The stiffness and the loss of autonomic control lead to trips and falls, especially when attempting to change direction, which subsequently leads to increased bone fractures and loss of confidence resulting in further reduced mobility.

Cognitive symptoms may occur early in the disease in patients without signs of dementia sometimes before the motor symptoms and before diagnosis and become more prevalent as PD progresses. Neuropsychiatric deficits in PD include executive dysfunction and impairment in language, memory and visual-spatial skills [10,11]. Executive dysfunction includes difficulties with planning, abstract thinking, and controlling inappropriate behaviors. PD patients often perform poorly on tasks requiring advanced planning, cognitive sequencing, and generation of metal sets [12,13]. Individuals with PD may also suffer from slowed processing speeds, meaning they are slow to answer questions, or recall memories, and have an impaired sense of time. These symptoms may be relieved, however, with visual cues [14]. Not all studies show language impairment in PD; however, there have been deficits identified in semantic fluency, verb naming and tasks of alternating fluency [15,16,17]. In nondemented PD patients, there have been reported deficits in visuospatial functions such as facial recognition, line orientation perception, or visual-motor construction [18]. Individuals affected with PD are six times more likely to suffer from dementia [19], which results in a significant reduction in quality of life for the patient, and difficulties for their caregivers and family members.

### 3.2. Epidemiology

PD is the second most common neurodegenerative disease (the first being AD), and the most common movement disorder around the world, with approximately 7 million people affected worldwide [20,21]. PD becomes more common in older age groups, with 1% of those 60 years and above, rising to 4% in those 80 and above [22,23]. The mean age of onset of PD symptoms is 60 years old, although early-onset PD symptoms may occur as young as 20 years old [24]. PD is usually sporadic; however, a genetic form of PD is thought to affect men more than women, with a ratio of 3:2 [25]. Various risk factors may apply to men more than women that explain the difference in risk associated with developing PD, although the link between them and developing PD cannot be seen as a cause and effect phenomena; instead they are associations or risk factors. These are thought to include severe head trauma [26], exposure to pesticides, dietary habits, as well as participating in various male-dominated occupations may also be associated with an increased risk of developing PD [27]. Conversely, caffeinated beverage drinkers appear to have a reduced risk of developing PD and, surprisingly, smokers are also less likely to suffer PD, although the link between these two risk factors has yet to be conclusively attributed to specific causes of PD [28].

### 3.3. Molecular Mechanisms and Causes

Heritable mutations account for less than 10% of all PD cases, which suggests that the primary causes are environmental exposure, with the addition of genetic vulnerability [29], along with significant risk factors from aging, being male, and traumatic brain injury. The loss of dopaminergic neurons is the central cause that links all forms of PD [30,31]. It is estimated that 80% of dopaminergic neuron loss occurs before any symptoms present [8]. While the initial causes of dopaminergic cell loss are not fully understood, the principal proposed causes of brain cell death in PD is the accumulation of oligomerized proteins, forming what is known as Lewy bodies. Lewy bodies are widely regarded as the cause of cell death or at least the beginning of a pathway that leads to cell death [32]. The Lewy bodies are formed from a protein called α-synuclein and are non-toxic until they form aggregates [33]. α-synuclein is linked with many pathways that can cause cell death, with roles in protein degradation and autophagy [34], endoplasmic reticulum stress [35], and histone acetylation [36].

One particularly significant effect of α-synuclein aggregation on the brain and PD is its effect on the mitochondria. Some of the α-synuclein expressed in the cell resides in the mitochondria, where it suppresses complex-I activity [37,38]. Complex-1 inhibition can subject the cell to oxidative stress and impair ATP production, leaving the cell without an energy source [39]. This localization at the mitochondria has drawn links between α-synuclein, mitochondrial toxins, and sporadic forms of PD [33]. The morphology of mitochondria is disrupted in transgenic mice expressing a mutant form of α-synuclein [40]. Many PD models implicate the role of oxidative stress, α-synuclein aggregation and mitochondria function, for example, transgenic mice overexpressing superoxide dismutase-1 are resistant to the oxidative stress caused by the neurotoxin MPTP compared to wild type mice [41].

Dopaminergic neurons are particularly subject to oxidative stress, due to the metabolism of dopamine, which produces H_2_O_2_ and superoxide molecules. Damage to the mitochondrial function and loss of energy production can disrupt the vesicles that store dopamine, causing dopamine-related oxidative reactions to occur in the cytoplasm. The increase in cytoplasmic oxidative stress causes misfolding of more proteins and aggregation of α-synuclein, leading to further damage to mitochondria and further release of reactive oxygen species (ROS) [42].

### 3.4. Treatments

There is no cure for PD; there are only treatments that manage the symptoms. None of these treatments can halt or reverse the progression of the symptoms. The principal treatment strategy is to replace the dopamine that is lost in the brain; however, dopamine does not pass the blood-brain-barrier (BBB). Therefore, dopamine is administered in the form of levodopa, which can pass the BBB and is metabolized into dopamine. Levodopa can alleviate the motor symptoms of PD and has been the preferred drug of choice for PD treatment for almost 40 years [43]. Only approximately 5–10% of levodopa passes the BBB, the remaining 90–95% is metabolized to dopamine elsewhere in the body, resulting in several side effects (gastrointestinal and cardiovascular) [44]. Some of these side effects may be prevented by administering inhibitors of the enzyme L-amino acid decarboxylase and catecholamine-*O*-methyltransferase (COMT) inhibitors, where the former prevents levodopa metabolism in the gut and improves its bioavailability. In contrast, the latter prevents the conversion of levodopa to 3-o-methyldopa [45]. Other treatments for PD include monoamine oxidase inhibitors (MAO-B inhibitors), which aim to reduce the breakdown of the available dopamine, and dopamine agonists, which are designed to mimic dopamine, with the ability to cross the blood-brain barrier (BBB). Despite these options for PD treatment, dopamine agonists and MAO-B inhibitors are inferior when it comes to treating motor symptoms and daily activities. Dopamine agonists tend to have more side effects but fewer motor complications [43]. PD would, therefore, benefit from new, more effective drugs that can slow or halt the progression of the disease.

## 4. Neuroprotective Properties of Green Tea against Parkinson’s Disease

### 4.1. Green Tea and α-Synuclein

α-synuclein is a 140 amino acid protein found in the brain and expressed predominantly in the presynaptic cleft of nerve cells. Usually, α-synuclein, with its alpha-helical structure, is involved in neuronal differentiation, regulation of dopamine synthesis, and suppression of apoptosis in neurons. Under physiological conditions, α-synuclein cannot form the fibrillar structure as there is an equilibrium existing between its monomeric and oligomeric form. Further, ubiquitin-proteasome machinery and lysosomal autophagic pathways ensure the removal of the excess and oligomeric α-synuclein in the healthy brain [46]. However, an increase in the level of α-synuclein, impaired mitochondrial function, and disruption in the association of α-synuclein with membrane increases the inclination of this non-toxic structure to form aggregates and disturb the normal mechanism in neurons leading to neuronal loss [47,48,49]. Hence one of the critical steps in preventing PD pathology is the prevention of aggregate formation.

In a cell-free in vitro system, green tea polyphenol epigallocatechin-3-gallate (EGCG) strongly inhibited the aggregation of α-synuclein and prevented the toxicity mediated through it in PC12 cells. EGCG could and favorably bind to native unfolded α-synuclein polypeptide chain and prevent the formation of toxic β structures by mediating the formation of the unstructured oligomer. It can also inhibit the addition of monomeric α-synuclein to fibrillar intermediates [50]. Epitope mapping on peptide membrane revealed that EGCG could bind with aggregation-prone sites through intermolecular hydrophobic interaction to prevent the fibrillization of α-synuclein [51]. Further, NMR studies show that EGCG could inhibit the transformation of α-synuclein to the β-sheet structure by binding with Ile, Phe, and Tyr residues. Furthermore, EGCG can disaggregate the preformed fibrillar structures into non-toxic amorphous protein structures, and this disaggregation may be due to the interaction of EGCG with Leu, His, Phe, and Tyr residues [52,53]. A study by Lorenzen and his group has shown that EGCG reduces the interaction between α-synuclein oligomers and cell membranes by significantly impeding with the oligomers and thereby protecting rat neuronal cells from toxicity [54].

### 4.2. Dopamine and Green Tea Extract

Parkinson’s disease mainly affects the Substantia Nigra pars compacta (SNpc) region in the brain, where dopaminergic neurons occur. It is predicted that <80% of the dopaminergic neurons are lost during the progression of PD, resulting in a decrease in the level and availability of dopamine in the brain [55]. Dopamine is one of the neurotransmitters, and under the normal physiological condition, it transmits the signals needed for the proper coordination of muscle movement by regulating the excitability of striatal neurons. However, loss of dopamine causes an irregular pattern of nerve firing and results in loss of movement control [56]. The neurotoxins 1-methyl-4-phenyl-1,2,3,6-tetrahydropyridine (MPTP), 6-hydroxydopamine (6-OHDA) selectively damages the substantia nigra region of the brain resulting in the dopaminergic neuronal loss and is widely used in identifying the underlying molecular mechanism in the progression of PD [57].

Treatment of catechin-rich tea polyphenol extract improved motor impairments, recovered tyrosine hydroxylase (TH), and dopamine level, reduced the level of α-synuclein oligomers, and their aggregation in cynomolgus monkeys administered with MPTP [58]. Pre-treatment with green tea extract and EGCG protected male C57/BL mice by reducing dopamine loss by modulating the striatal antioxidants, superoxide dismutase (SOD), and catalase to significant levels. Further, they prevented the reduction of TH, the enzyme that catalyzes L-dihydroxyphenylalanine (L-DOPA) formation from tyrosine in the biosynthetic pathway of dopamine and thereby prevented the dopaminergic neuronal death [59]. EGCG pre-treatment to C57/BL mice administered with MPTP reduced the expression of α-synuclein and prevented apoptosis of neurons by increasing and decreasing the expression of Bcl-2 and Bax respectively. Further, it also offered neuroprotection via increasing the level of protein kinase C-α (PKC-α) in the striatum [60].

**Figure 2:**
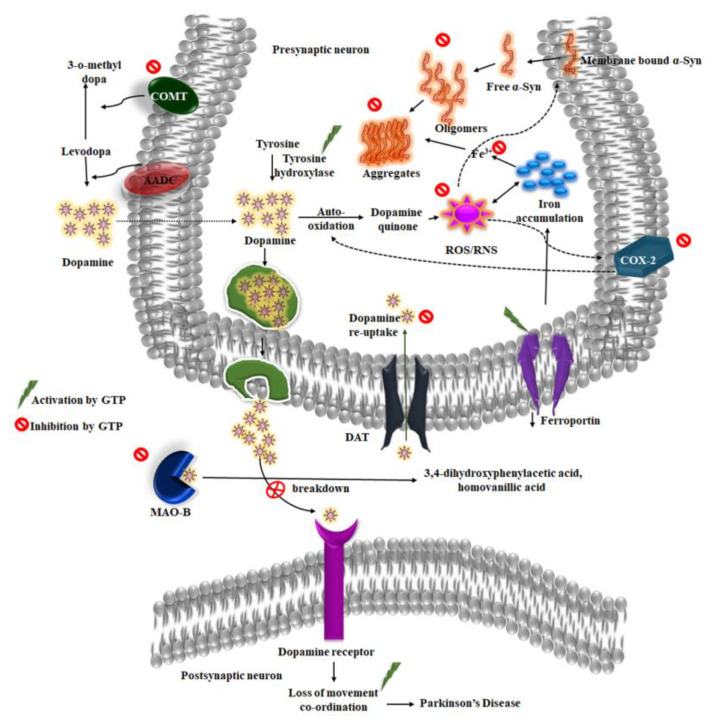
Neuroprotective action of green tea polyphenols (GTP’s) in prevention of the pathological process occurring during Parkinson’s disease. GTP can possibly act as inhibitors of α-synuclein aggregation, MAO-B, COMT, prevent the accumulation of iron and activate TH to protect dopaminergic neurons degeneration.

EGCG also induced PKC expression in mediating the degradation of the pro-apoptotic protein Bad to offer neuroprotection in human NB SH-SY5Y cells [61]. PKC’s activity is involved in neuronal survival mechanisms activating several protein phosphorylation pathways including ERK and JNK [62]. Additionally, EGCG inhibited the ability of dopamine transporters (DAT) to actively uptake MPP^+^ and being transported to presynaptic dopaminergic neurons and thereby prevented neurodegeneration in CHO cells expressing DAT [63]. Under physiological conditions, DAT is involved in the reuptake of dopamine from the synaptic cleft and terminate the signaling. However, in PD, since the availability of dopamine in the nerve terminals is reduced, inhibiting DAT is one approach to prevent disease progression [64]. The study by Li et al. showed that EGCG without affecting DAT expression induces its internalization through the activation of PKC in DAT-PC12 cells, thereby preventing the reuptake of dopamine from the synaptic cleft [65]. Long-term use of L-DOPA for PD treatment shows adverse effects that might be due to the formation of 3-O-methyl dopa, which would further decrease dopamine turnover and inhibit dopamine transporter and uptake. Catecholamine-O-methyltransferase (COMT) inhibitors prevent the conversion of L-DOPA to 3-O-methyl dopa and are used in combination for PD treatment. EGCG has been shown to inhibit COMT both under in vitro and in vivo conditions, which could effectively prevent further methylation of L-DOPA (Figure 2), suggesting that EGCG can also be given in combination along with the existing drugs to improve their availability and efficiency in the brain [66].

### 4.3. Inhibition of MAO-B

The enzyme, monoamine oxidase-B (MAO-B), is primarily seen in the brain and is involved in the enzymatic conversion of dopamine to 3,4-dihydroxyphenylacetic acid and homovanillic acid [67]. An increase in the MAO-B level has been found in the aging brain that plays a significant role in PD, and inhibition of MAO-B enzymatic activity results in the inhibition of dopamine breakdown and increases its availability in the synaptic cleft [68]. EGCG, apart from enhancing the conversion of dopamine, is also involved in the inhibition of MAO-B in aged rat brain, indicating its multipotential role against PD [69].

### 4.4. Iron and Green Tea

The accumulation of iron in the brain’s substantia nigra region is thought to be one of the pathological implications in PD. Under physiological conditions, iron plays a significant role in maintaining the functions of the cell. However, during pathological conditions, there is an iron increase in the brain due to BBB disruption or a fault in the iron storage and transport mechanisms. The accumulated iron, in turn, could induce oxidative stress, aggregation of α-synuclein, neuroinflammation, and contribute to the degeneration of neuronal cells [70]. The transition metal form of iron, Fe^3+^, is majorly involved in promoting the aggregation of α-synuclein, chelation of iron is considered one of the therapeutic strategies for PD treatment. Fe^3+^ can interfere with Tyr and Ala residues of α-synuclein and accelerate the rate of fibrillization.

However, EGCG treatment chelates Fe^3+^ and prevents the fibrillation and toxic oligomer formation in a cell-free in vitro system [71]. Additionally, binding assay studies show that EGCG could inhibit the chemical reactivity of Fe^2+^ in Fenton reaction by forming Ngal–EGCG–iron complex and thereby prevent continuing aggravation of free radicals [72]. The iron-related proteins hepcidin-ferroportin plays a significant role in maintaining iron homeostasis in the brain. It has been demonstrated that MPTP treatment reduces the expression of ferroportin, thereby accumulating iron in the brain. EGCG treatment offered neuroprotection to C57 mice administered with MPTP by increasing motor co-ordination, dopamine concentration, and ferroportin expression [73]. In compliance with the notion that iron chelators could also induce differentiation, EGCG induced neurite outgrowth, and neuronal differentiation in SN4741 cells by downregulating cell cycle proteins cyclin D1, E [74]. Iron chelation therapy is considered as a strategy for the treatment of PD. The ability of green tea polyphenols to act as iron chelators along with other neuro-rescue properties may offer a better chance of alleviating PD.

### 4.5. Antioxidant Potential of Green Tea in Combating Oxidative Stress

Presently, significant emphasis is given to oxidative stress in any chronic disease management. With no exemption, oxidative stress plays a major role in neurodegenerative diseases as the brain is highly vulnerable to such stress because of the presence of a high amount of lipid content and oxygen consumption. Reactive oxygen species (ROS) have a strong potential to oxidize the biological macromolecules and cause detrimental effects [75]. Hence, a combinatorial approach of drugs along with antioxidants have been suggested for combating them. Dietary supplementation of antioxidants has been associated with ameliorating the pathological outcomes. Green tea, due to the presence of a high amount of antioxidants, can counteract the detrimental effects of oxidative stress either directly or by interfering with the host’s defense mechanism. The antioxidant activity of the green tea polyphenols is due to the hydroxyl groups presence in the chemical structure which could donate electrons and/or H^+^ for the neutralization of free radicals [76].

Green tea polyphenols also have positive effects on the antioxidant enzymes. Consumption of green tea by PD affected individuals showed a marked increase in the antioxidant enzymes catalase, SOD, and reduced the oxidation of proteins and lipids [77]. Epicatechin gallate significantly restored the locomotor activity and reduced lipid peroxidation, oxidative stress-mediated apoptosis in the mutant Drosophila model of PD expressing α-synuclein [78]. Oxidation of proteins and lipids contributes to the degeneration of dopaminergic neurons, which could be corroborated by the presence of oxidation by-products protein carbonyl and 4-hydroxyl-2-nonenal (4-HNE) in high concentration in the postmortem brain of individuals with PD [79,80].

Lipid peroxidation also causes structural degeneration of the membrane, induces apoptosis, and free reactive oxygen generates further radicals that continue the chain reaction [81]. HNE can interact effectively with α-synuclein and make modifications by forming covalent adducts, which form toxic stable oligomers [82]. A study on Portuguese subjects on the effect of drinking green tea showed a significant reduction in malondialdehyde levels (MDA) and 4-HNE, the lipid oxidation products [83]. L-theanine, a major free amino acid component of green tea, protected a co-culture containing primary neuronal and neuron-astrocyte cells from excess dopamine-induced toxicity by inducing glutathione (GSH) supply in striatal astrocytes [84]. GSH is one of the main antioxidant molecules and increasing the neuronal GSH level is considered one of the therapeutic strategies for treating neurodegenerative conditions [85]. Transcriptional upregulation of GSH in glial cells was found to protect the neuronal cells from oxidative stress-induced by 6-OHDA and H_2_O_2_, which implicates the importance of GSH in PD treatment [86].

Glutathione depletion in cells leads to ROS generation, which further elicits apoptotic signals [87]. Experimental results from CGN cultures indicates that the green tea polyphenol EGCG protects the cells from mitochondrial oxidative stressors by selectively localizing in the mitochondria displaying intrinsic targeting and protecting neurons from apoptosis, a mechanism by which it could interfere with the free radicals being formed and maintain the normal homeostasis condition [88]. Green tea polyphenol treatment in 6-OHDA rat PD models showed neuroprotective effect by the inhibition of ROS, RNS species, and peroxidation of lipids, reducing iNOS and protein-bound 3-nitro-tyrosine level (3-NT) [89]. Nitrite radicals formed during stress conditions can react with tyrosine residues in the protein to form protein-bound 3-NT, which can be seen in several neurodegenerative diseases, including PD [90]. Lewy bodies seen in PD contains an increased amount of nitrated tyrosine residues and α-synuclein, the major constituent of Lewy bodies has also been found to be nitrated in animal models of PD and postmortem PD brains [91]. Additionally, 3-NT modification has been found to inhibit the binding of α-synuclein to synthetic vesicles reducing its degradation rate and increasing fibril formation [92]. The reduction of 3-NT by green tea polyphenol indirectly increases the degradation rate of α-synuclein, implying its promising role in combating PD. In knockdown dj-1-β flies, paraquat exhibited impaired locomotion, increased oxidative stress, and neurodegeneration. The protein DJ-1 is involved in antioxidant activity, and it has been reported to exhibit a protective effect in flies by modulating Daxx/JNK/Drosophila FOXO pathway. EGCG was found to protect the mutant flies from paraquat-induced oxidative stress and improved motor function [93].

### 4.6. Green Tea in Alleviating Mitochondrial Dysfunction

Mounting evidence is indicating the role of mitochondrial dysfunction in the pathogenesis of PD [94]. Though motor deficit is the major outcome of PD, several pathological events, including mitochondrial dysfunction, occur earlier before the clinical symptoms become prominently visible. Normally, α-synuclein gets localized in mitochondrial associated membranes, thereby forming an interface between mitochondria and endoplasmic reticulum that helps in the regulation of Ca^2+^ signaling and apoptosis. An increase in the expression of α-synuclein or the presence of its aggregated form causes dissociation of mitochondria and ER, which further results in the loss of Ca^2+^ signaling and decrease in mitochondrial energy production [95]. The impaired mitochondrial function may be due to the ability of α-synucleins to cause mitochondrial permeability transition pore (mPTP) formation that further causes loss of mitochondrial membrane potential (MMP) resulting in mitochondrial degradation and ultimately cell death [96]. Hence, small molecules that can maintain the mitochondrial function and interfere with signaling pathways will be of great use in combating PD.

Green tea polyphenol EGCG has shown protection to SH-SY5Y cells from 6-OHDA induced neurotoxicity by suppressing the buildup of ROS, restoring MMP, and maintaining calcium homeostasis [97]. Additionally, EGCG protected PC12 cells from Oxy-Hb induced stress by restoring MMP, preventing the opening of mPTP, and eventually the release of cytochrome-c. It also protected mitochondrial function by inhibiting Ca^2+^-influx through voltage-gated calcium channels and mitochondrial Ca^2+^-uptake by mitochondrial Ca^2+^ uniporter [98].

**Figure 3:**
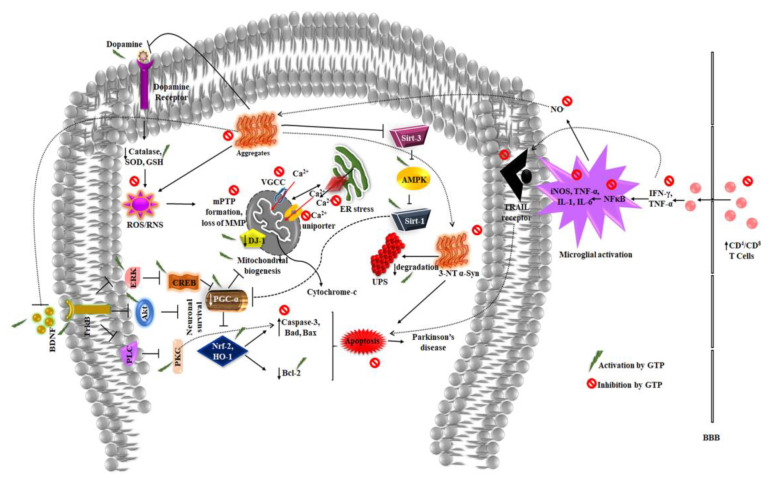
GTP’s role in inducing mitochondrial biogenesis, activating stress response genes and suppressing neuroinflammation, inhibiting oxidative stress, and apoptosis of dopaminergic neurons and protecting from the pathological changes occurring during Parkinson’s disease (VGCC—Voltage gated calcium channel; UPS—Ubiquitin proteosome system).

Peroxisome proliferator-activated receptor γ coactivator-1 α (PGC-1α) induces the expression of several genes responsible for mitochondrial biogenesis, antioxidant detoxifying enzymes, and is regarded as the ‘master regulator’ [99]. Binding of CREB on PGC-1α gene induces its expression, which causes the activation of Nuclear factor erythroid 2-related factor 2 (Nrf-2) and heme oxygenase-1 (HO-1) to protect cells from oxidative stress [100]. PGC-1α expression is also mediated by SIRT-1, which deacetylase the protein to enhance its activity [101]. The expression of PGC-1α is found to be low in PD condition, and the overexpression of the same has been reported to protect dopaminergic neurons in PD cellular models, making it a key target in the therapeutic intervention of PD. In PC12 cells, EGCG treatment exerted a neuroprotective effect against MPTP toxicity by the activation of PGC-1α via SIRT-1 signaling [102,103]. Long-term administration (6 months) of EGCG in C57BL/6 J mice prevented age-related cognitive decline and improved locomotor activity by increasing the expression of CREB and post-synaptic proteins PSD95, CAMKII [104]. Activation of CREB by green tea polyphenols exerts multifaceted benefits like regulating mitochondrial biogenesis via PGC-1α, induce neurogenesis, and cellular survival to protect dopaminergic cells (Figure 3).

Exposure to environmental toxicants like rotenone and dieldrin is also associated with the risk of developing PD. L-theanine showed a neuroprotective effect on SH-SY5Y cells from rotenone and dieldrin induced toxicity by downregulating HO-1, caspase-3, and inducing the release of neurotrophic factors BDNF and GDNF [105]. HO-1 is a stress response protein and whose presence in larger quantities is corroborated with its attempt to bring back the normal redox status in the respective tissue [106]. The downregulation of HO-1 by L-theanine indicates that the non-proteinogenic amino acid can significantly suppress the oxidative stress and maintain the normal homeostasis to exert neuroprotection.

### 4.7. Activation of Neurotrophic Factors and Signaling Pathways

Several bioactive molecules have been shown to exhibit their activity by modulating the intracellular pathways [107]. L-theanine showed an increase in the expression of ERK1/2 to protect SH-SY5Ycells from neurotoxicity [105]. ERK1/2 pathway mediates dopaminergic signaling and maintains the activity of striatal neurons. It is also involved in learning, memory, and neuronal development [108,109]. Understanding the signaling mechanism through which green tea polyphenol exerts neuroprotection could also help develop of new therapeutic approaches.

Brain-derived neurotrophic factor (BDNF) is a major neurotrophic factor involved in the neurogenesis process, and its decreased expression has been associated with impaired motor performance in individuals with PD. BDNF exerts its activity by binding with the cell surface receptor tyrosine receptor kinase B (TrkB), and the subsequent phosphorylation and activation lead to the cell survival mechanism by further activating many intracellular pathways, including MAPK, Akt, and PLC signaling [110]. In the PD condition, α-synuclein has been found to interact with TrkB and inhibit its function, contributing to the death of dopaminergic neurons [111]. Additionally, the brains of individuals with PD show a decreased level of BDNF, indicating its neuroprotective function [112]. Hence stimulating the BDNF level by small molecules is an effective treatment strategy in combating PD. A combination of rasagiline and EGCG was found to restore C57/BL6 mice from MPTP induced parkinsonism by increasing the dopamine level, inducing the expression of BDNF, phosphorylated PKC-α as well as Ras and its downstream effector Akt [74].

Similarly, EGCG treatment protected C57/BL6 mice from sevoflurane-induced apoptosis by regulating the expression of BDNF-TrkB and activating Akt signaling [113]. An increase in expression of BDNF to promote neurogenesis by EGCG may be due to its ability to activate CREB, as BDNF is one of the downstream targets for CREB [114]. Studies have indicated that Akt signaling is essential in neuronal survival by inducing the release of several neurotrophic factors. Additionally, reduced Akt signaling has been observed in the post-mortem PD brain compared to the non-PD brain [115]. Acetylated EGCG has been reported to ameliorate 6-OHDA induced toxicity in differentiated SH-SY5Y cells by acting through the Akt signaling pathway and prevented apoptosis by downregulating caspase-3 activity [116].

### 4.8. Neuroinflammation

Several reports have pointed towards the chronic inflammatory processes occurring in SNpc of PD brains, causing the death of dopaminergic neurons. Though central nervous system (CNS) has been regarded as an immune-privileged site, during PD due to the breakdown of BBB integrity, the innate immune system is activated, which in turn secretes and triggers a series of inflammatory events including cytokines, ROS and RNS leading to the progression of PD. Upon degeneration of dopaminergic neurons, peripheral immune cells enter brain parenchyma, where they come in contact with microglial cells that further exacerbates the inflammatory condition. During this process, the lymphocyte populations have been reported to alter individuals with PD and the PD rat model [117]. EGCG was found to modulate peripheral immunity by reducing CD4+ to CD8+ ratio and downregulating the expression of TNF-α, IL-6, thereby offering neuroprotection against MPTP-induced toxicity in C57BL/6 J mice [118]. Neuroinflammation occurring during PD induces the expression of inflammatory mediators like TNF-α and interleukins and plays a major role in the initiation of apoptosis. In the rotenone-induced rat PD model, EGCG treatment significantly downregulated the expression of TNF-α, IL-1, IL-6, and showed a neuroprotective effect [119].

TNF-related apoptosis-inducing ligand (TRAIL) belongs to the tumor necrosis factor (TNF) superfamily and it is involved in evoking apoptosis via death receptors. Though the intrinsic apoptotic pathway mediated cell death is observed, death receptor-mediated apoptosis has also been evidenced in PD. EGCG has been found to inhibit the expression of TRAIL ligand and TRAIL receptor DR5 in SH-SY5Y neuroblastoma cells [120]. Although TRAIL expression is comparatively low in the CNS, the pro-inflammatory cytokine interferon-γ can induce its expression [121]. An elevated level of IFN-γ has been observed in the blood plasma of PD affected individuals, highlighting the relationship between TRAIL and IFN-γ [122]. Liu et al., have reported the neuroprotective property of EGCG by suppressing IFN-γ in LPS induced toxicity in human macrophages [123]. The inhibition of death receptor-mediated apoptosis by EGCG may be due to its inhibitory activity on IFN-γ, which, in turn, suppressed TRAIL activity.

Treatment with a standardized extract of green tea and its active components epicatechin and EGCG reduced the oxidation capacity and reversed a drastic increase in the level of inflammatory mediators COX-2 and iNOS from 6-OHDA induced neurotoxicity in male Wistar rats [124]. Though iNOS is expressed minimally in the brain compared to nNOS, during pathological conditions of PD, there is a sharp increase in both of their level in the SNpc region and contributes to the production of NO that causes degeneration of dopaminergic neurons. Inhibition of iNOS and nNOS by EGCG has also been observed in the MPTP mouse model of PD. This could be due to the direct inhibition of EGCG to uptake MPP^+^ or inhibiting iNOS transcription by averting the binding of NFκB to the iNOS promoter [125,126,127,128]. Additionally, there is compelling evidence that iNOS can induce the expression of COX-2 and vice versa suggesting inflammatory processes are associated with PD. In the postmortem brain of PD specimens, increased expression of COX-2 and iNOS is seen to support the notion. Selective inhibition of COX-2 further prevents the formation of oxidation species, dopamine-quinone, and, thereby, protection from dopaminergic neurodegeneration [129,130,131,132].

The transcription factor NF-κB controls the expression of many inflammatory genes and is regarded as the master switch required for their expression [133]. Hence, targeting and switching off the NF-κB activity has emerged as a therapeutic option in the treatment of PD. Green tea extract has been found to protect neuronal cultures from 6-OHDA-induced toxicity by averting nuclear translocation and activation of NF-κB [134].

### 4.9. Alteration of the Gut Microbiome

EGCG treatment to PINK1 null mutant PD flies rescued them from the motor and neuronal deficits. Further, gut microbiota was found to be associated with EGCG mediated neuroprotective effect supporting the interlink between the microbiome-gut-brain axis and PD. Several studies have pointed out the imbalanced gut microbiome and differential microbiota in PD affected individuals compared with healthy people [135,136,137]. EGCG treatment in flies significantly altered the microbial profile in PD models and brought them back to a comparable profile as that of control indicating intestinal microbiome compositions are being reshaped by the phytochemical. The stress response protein TotM was found as the central gene involved in EGCG-mediated neuroprotection by responding to gut microbiota signals and rescued motor deficits and dopaminergic neuron morphology [138].

The activity of green tea and its polyphenols against PD with in vitro and in vivo evidences explained in this section have been represented in Table 1 and Table 2 respectively.

## 5. Future Perspective and Conclusions

The idea to elicit neuroprotection and delay aging and age-related diseases through natural sources is on the rise because of the limited side effects. Different diet patterns, mainly the Mediterranean and Asian diets, which include the consumption of different polyphenols, is gaining more attraction due to the health benefits they offer, including neuroprotection [139]. Still, the polyphenols should be consumed in the optimal dose to avail maximum benefits for the host. Despite the emerging data on the neuroprotective effect of green tea against PD, consideration must be given to the dosage and safety aspect of its consumption. Though most of the reports point out that green tea extract is devoid of mutagenicity, genotoxicity and safe up to 2000 mg/kg b.w. in rodents based on toxicity studies, there are also reports which hint hepatotoxicity and damage to gastro-intestinal (GI) tract [140,141,142,143]. However, a meta-analysis on the published toxicology and human intervention studies indicates that the mode of consumption and dosing conditions play a major role in exerting the toxic effect. The study shows that green tea extract and EGCG administered along with the dietary route or drinking water did not show any detrimental effects even after long term exposure.

Moreover, green tea extract fed under fasting conditions or using preparations in a concentrated manner has shown hepatotoxicity and damage to the GI tract. Further, in beverage form 704 mg EGCG/day and as concentrated solid bolus dose 338 mg EGCG/day was considered to be an observed safe level under fed conditions in adults [144]. Despite these reports, there is a possibility that the detrimental events caused by green tea may be under-reported, and thus, more caution and more clinical studies may be appropriate.

Another major hurdle for PD treatment is the inefficient delivery of polyphenols into the brain due to the BBB [145]. The ability of EGCG and its metabolites to reach the brain parenchyma through the blood-brain barrier and induce neuritogenesis have been identified in an in vitro study [146,147]. Although few studies hints on the BBB penetration of green tea polyphenols and pre-clinical trials show promising results, the lack of solubility and reduced availability may be possible reasons for not having many successful clinical studies in humans [148,149]. Structural modification of green tea polyphenols, combined with nano delivery, could be the next milestone in treating neurological diseases, including PD [150]. However, the efficiency and compatibility of tea polyphenols for this approach have to be closely monitored.

To conclude, green tea polyphenols have been found to have various pharmacological actions against PD by modulating several genes’ expressions and interfering with signaling pathways. Though the outcome of the reports reviewed seems to be very promising, there are still certain obstacles being faced in developing it into a complete drug because of its solubility, bioavailability, penetration across the BBB, and limited clinical studies. An interdisciplinary approach of various fields is needed so that the chemical modification strategy or nano-drug delivery mechanisms can be introduced to enhance its activity.

## Figures and Tables

**Table 1:** Green tea can exhibit anti-Parkinson’s effect—*in vitro* evidence.

Sl. NO.	Model Used	Mode of Action	Reference
1	Cell free in vitro system	EGCG inhibits the aggregation of and induces disaggregation of α-synuclein	[50,51,52,53,54]
2	PC12 cells	EGCG protected PC12 cells from α-synuclein induced toxicity	[50]
3	AS-PC12 cells	EGCG protected AS-PC12 cells from Fe(III) induced toxicity by reducing the formation of ROS	[52]
4	NB SH-SY5Y cells	EGCG offered neuroprotection by PKC mediated degradation of pro-apoptotic protein Bad	[61]
5	CHO cells expressing DAT	EGCG prevented neurodegeneration by inhibiting the ability of dopamine transporters (DAT) to actively uptake MPP+ and being transported to presynaptic dopaminergic neurons	[63]
6	DAT-PC12 cells	EGCG induces the internalization of DAT through the activation of PKC thereby preventing the reuptake of dopamine from the synaptic cleft	[65]
7	Cell free in vitro system	EGCG treatment chelates Fe^3+^ and prevents the fibrillization and toxic oligomer formation	[71]
8	SN4741 cells	EGCG downregulates cell cycle proteins cyclin D1, E and helps in neurite outgrowth and neuronal differentiation	[74]
9	Primary neuronal and neuron-astrocyte cells	L-theanine, protected cells from dopamine-induced toxicity by inducing glutathione production	[84]
10	CGN culture	EGCG inhibited apoptosis of neuronal cultures from mitochondrial oxidative stressors	[88]
11	SH-SY5Y cells	EGCG protected cells from 6-OHDA induced neurotoxicity; suppressing the buildup of ROS, restoring MMP, and maintaining calcium homeostasis	[97]
12	PC12 cells	EGCG protects cells from Oxy-Hb induced stress; inhibits Ca^2+^-influx through voltage-gated calcium channels	[98]
13	PC12 cells	EGCG protected cells from MPTP toxicity by the activation of PGC-1α via SIRT-1 signaling	[102]
14	SH-SY5Y cells	L-theanine exhibited neuroprotective effect against rotenone and dieldrin toxicity by downregulating HO-1, caspase-3, inducing neurotrophic factors BDNF and GDNF and activating ERK1/2 pathway	[105]
15	SH-SY5Y cells	EGCG ameliorates 6-OHDA toxicity via Akt signaling pathway and prevents apoptosis by downregulating caspase-3 activity	[116]
16	SH-SY5Y cells	EGCG inhibits TRAIL ligand expression as well as TRAIL receptor DR5	[120]
17	Macrophage cells	EGCG protects macrophage cells from LPS induced toxicity by inducing the expression of IFN-γ	[123]
18	Neuronal cultures	Green tea extract attenuated 6-OHDA induced NF-κB activation and cell death	[134]

**Table 2:** Green tea can exhibit anti-Parkinson’s effect—*in vivo* evidence.

Sl. No.	Model Used	Activity Observed	Reference
1	Cynomolgus monkeys	Catechin-rich tea polyphenol extract improved motor impairments and restored TH and dopamine levels in MPTP PD model.	[58]
2	C57/BL mice	Green tea extract and EGCG reduced the loss of dopamine by modulating the antioxidant enzymes in MPTP PD model.	[59]
3	C57/BL mice	In MPTP PD model EGCG reduced the expression of α-synuclein and prevented apoptosis by downregulating the expression of Bax and increasing the expression of PKC-α	[60]
4	Long-Evans Rats	EGCG inhibited MAO-B in aged rat brain	[69]
5	C57 mice	EGCG induced ferroportin expression and offered neuroprotection	[73]
6	PD affected individuals	Green tea consumption showed a marked increase in the antioxidant enzymes catalase, SOD, and reduced the oxidation of proteins and lipids	[77]
7	Drosophila	Epicatechin gallate restored locomotor activity and reduced lipid peroxidation, oxidative stress	[78]
8	Human	Green tea exerts beneficial effect, by reducing oxidative stress and protects the individual against oxidative stress diseases	[83]
9	Sprague-Dawley Rats	Green tea polyphenol exhibits neuroprotective effect against 6-OHDA by reducing lipid peroxidation, 3-NT level.	[89]
10	Knockdown dj-1-β Drosophila	EGCG prevented oxidative stress and neurodegeneration induced by paraquat.	[93]
11	C57BL/6J mice	Long-term administration of EGCG prevented age-related cognitive decline and improved locomotor activity by increasing the expression of CREB and post-synaptic proteins PSD95, CAMKII.	[104]
12	C57/BL6 mice	A combination of Rasagiline and EGCG restored mice from MPTP induced parkinsonism by increasing the expression of BDNF, phosphorylated PKC-α as well as Ras and its downstream effector Akt	[74]
13	C57/BL6 mice	EGCG protects from sevoflurane by regulating the expression of BDNF-TrkB and activating Akt signaling	[113]
14	C57BL/6J mice	EGCG reduced CD4+ to CD8+ ratio downregulating the expression of TNF-α, IL-6 in MPTP treated mice	[118]
15	Male Wistar Rats	EGCG reduced rotenone induced parkinsonism like symptoms in rats by downregulating the expression of TNF-α, IL-1, IL-6	[119]
16	Male Wistar rats	Standardized green tea extract and its active constituents downregulated the expression of inflammatory mediators COX-2 and iNOS by 6-OHDA	[124]
17	C57BL/6 mice	EGCG inhibited iNOS expression and cell death induced by MPTP	[125]
18	PINK1 null mutant Drosophila	EGCG rescued flies from motor, neuronal deficits and significantly remodeled gut microbiota	[138]

## Abbreviations

PD	Parkinsons disease
AD	Alzheimer’s disease
HD	Huntington’s disease
ROS	Reactive oxygen species
MAO-B	Monoamine oxidase B
BBB	Blood brain barrier
EGCG	Epigallocatechin-3-gallate
SNpc	Substantia Nigra pars compacta
MPTP	1-methyl-4-phenyl-1,2,3,6-tetrahydropyridine
6-OHDA	6-hydroxydopamine
TH	Tyrosine hydroxylase
SOD	Superoxide dismutase
L-DOPA	L-dihydroxyphenylalanine
PKC-α	Protein kinase C-α
DAT	Dopamine transporters
COMT	Catecholamine-O-methyltransferase
4-HNE	4-hydroxyl-2-nonenal
MDA	Malondialdehyde
GSH	Glutathione
3-NT	3-Nitrotyrosine
mPTP	Mitochondrial membrane permeability transition pore
MMP	Mitochondrial membrane potential
PGC-1α	Peroxisome proliferator-activated receptor γ coactivator-1 α
TrkB	Tyrosine receptor kinase B
CNS	Central nervous system
TRAIL	TNF-related apoptosis-inducing ligand
